# Do detour tasks provide accurate assays of inhibitory control?

**DOI:** 10.1098/rspb.2018.0150

**Published:** 2018-03-28

**Authors:** Jayden O. van Horik, Ellis J. G. Langley, Mark A. Whiteside, Philippa R. Laker, Christine E. Beardsworth, Joah R. Madden

**Affiliations:** Centre for Research in Animal Behaviour, Psychology, University of Exeter, Exeter, UK

**Keywords:** executive function, executive control, Cylinder task, Barrier task, cognition

## Abstract

Transparent Cylinder and Barrier tasks are used to purportedly assess inhibitory control in a variety of animals. However, we suspect that performances on these detour tasks are influenced by non-cognitive traits, which may result in inaccurate assays of inhibitory control. We therefore reared pheasants under standardized conditions and presented each bird with two sets of similar tasks commonly used to measure inhibitory control. We recorded the number of times subjects incorrectly attempted to access a reward through transparent barriers, and their latencies to solve each task. Such measures are commonly used to infer the differential expression of inhibitory control. We found little evidence that their performances were consistent across the two different Putative Inhibitory Control Tasks (PICTs). Improvements in performance across trials showed that pheasants learned the affordances of each specific task. Critically, prior experience of transparent tasks, either Barrier or Cylinder, also improved subsequent inhibitory control performance on a novel task, suggesting that they also learned the general properties of transparent obstacles. Individual measures of persistence, assayed in a third task, were positively related to their frequency of incorrect attempts to solve the transparent inhibitory control tasks. Neophobia, Sex and Body Condition had no influence on individual performance. Contrary to previous studies of primates, pheasants with poor performance on PICTs had a wider dietary breadth assayed using a free-choice task. Our results demonstrate that in systems or taxa where prior experience and differences in development cannot be accounted for, individual differences in performance on commonly used detour-dependent PICTS may reveal more about an individual's prior experience of transparent objects, or their motivation to acquire food, than providing a reliable measure of their inhibitory control.

## 1. Introduction

In humans, executive functions aid one's ability to monitor and control thoughts and actions [1]. Central to these processes are capacities for intentional, goal-directed behaviours and the ability to inhibit prepotent responses. Attempts to quantify such capacities are becoming a popular measure of cognitive ability in a variety of non-human animals, for example in birds [2–6], dogs [7–12], wolves [12], monkeys [13], apes [14,15] and fish [16]. One class of tasks, adopted from work on human infants [17], are particularly prevalent. These tasks require subjects to detour around a transparent obstacle, such as a barrier or cylinder, to obtain a food reward (for review see [18]). Capacities for inhibitory control are inferred when subjects cease making redundant attempts to directly acquire a visible reward that is made inaccessible by a transparent obstacle, and instead detour around the transparent obstacle to obtain the reward.

Recent work has indicated that interpreting performance on detour tasks is not straightforward. In a comparative study involving 567 individuals from 36 species of birds and mammals, MacLean and colleagues [19] used the Cylinder task and a complementary ‘A-not-B’ task to find that capacities for inhibitory control were higher in species with a large absolute, rather than relative, brain size; particularly anthropoid primates with a wide dietary breadth. However, subsequent more detailed species-specific work has cast doubt on the interpretation of these findings (see [3]). Ravens (*Corvus corax*), New Caledonian crows (*C. moneduloides*) and Jackdaws (*Corvus monedula*) have recently demonstrated comparable performances to the apes from the MacLean *et al*. [19] study on the Cylinder task [5], and New Caledonian crows show improved performance on an A-not-B task if they were trained to attend to an experimenter's hand movements [3], despite substantially smaller absolute brain sizes. Yet parrots showed poor performances on this task, despite their relatively large brain size [4], and guppies (*Poecilia reticulata*) showed comparable performances to most of the birds and mammals reported in MacLean *et al*. [19] despite their much smaller brain size [20]. Accordingly, the relationship between absolute brain size and capacities for inhibitory control remains unclear. Furthermore, these subsequent studies suggest that an individual's performance on detour tasks may fail to reflect their purported cognitive capacities as they are influenced by processes that are unrelated to inhibitory control.

There is growing evidence to suggest that individual performances on different tasks believed to reveal inhibitory control fail to accurately measure the same cognitive process or mechanism. For example, no individual consistency across different inhibitory control tasks has been found in dogs [8,10,11]. Such findings question the construct validity of inhibitory control tasks and thus the cognitive mechanism underpinning performances on these tasks. Consequently, we hereafter refer to such tasks as Putative Inhibitory Control Tasks (PICTs). Accordingly, a number of non-cognitive traits may contribute to differences in performances on cognitive tasks [21,22]. For example, North Island robins (*Petroica longipes*) in poor Body Condition showed impaired performances on an inhibitory control task compared with those in good Body Condition [23]. Moreover, increasing arousal has been found to enhance inhibitory control performances in calm, but not excitable, dogs [9]. Hence numerous non-cognitive traits may influence performance on inhibitory control tasks.

As the prior test history of subjects are rarely reported, it also remains possible that successful performances are facilitated by an individual's past experience. This may be particularly relevant when comparing performances of long-lived, enculturated, species, like the apes tested in the MacLean *et al*. [19] study, with those of wild-caught species that may have less exposure to testing (i.e. [3]). Apes in captivity, for example, may engage in a wide variety of cognitive tasks throughout their lifetime and are typically tested by humans, which interact with them behind transparent barriers [24]. Yet, sanctuary chimpanzees and bonobos have been found to outperform their zoo counterparts on an inhibitory control task [15]. Comparisons of inhibitory control performance in domestic species, such as dogs [8], may also be confounded by different experiences with transparent barriers, such as glass windows and doors. Such experiences may explain why pet dogs outperformed shelter dogs on an A-not-B task, although no differences were found in the Cylinder task [11]. It therefore remains important to explicitly test whether different experiences with transparent barriers can influence inhibitory control performance.

Pheasants, *Phasianus colchicus*, present a unique opportunity to negate these confounds. Pheasants are precocial and if artificial incubators are used, batches of individuals can be hatched on the same day, to control for age effects. They can be reared under standardized conditions to control for prior exposure to, for example, transparent barriers. Pheasant chicks are food motivated and readily engage with novel test apparatuses [22], and have previously been tested with a motor physical control task [25]. Pheasants, like other bird species assayed in studies of inhibitory control [19], might be expected to exhibit inhibitory control in a range of natural contexts including appropriate choice of food, responses to predators, and movement in the landscape. Pheasants were presented with two different PICTs, a Cylinder task and a Barrier task, but were divided into two groups so that they experienced these tasks in a counterbalanced order. For each task, birds were required to successfully navigate a pre-training apparatus, in which they learned to detour around an opaque apparatus to acquire a mealworm reward. Birds were then presented with an identical, but transparent, test apparatus in which they could similarly obtain a clearly visible mealworm. Performances presumably indicative of inhibitory control were quantified by recording subjects' latencies to acquire the reward worm, as well as the of number times they incorrectly pecked at mealworms through a transparent barrier, rather than detouring around the barrier. If previous experience with transparent barriers influences subsequent performance on PICTs, we predicted birds would show reduced latencies to solve, and make fewer incorrect attempts to acquire the reward on a second, albeit novel, task. Hence, we used a between-subjects approach to address whether birds that began with the Cylinder task and proceeded to the Barrier task showed superior performances on the Barrier task (presented second), compared with those birds that began with the Barrier task and proceeded to the Cylinder task (and vice versa).

To further investigate the relationship between dietary breath and performance in PICTs, as found in primates [19], we also conducted a free-choice ‘dietary breadth’ task, in which birds could sample a variety of familiar food items within a standardized time-frame. As motivational, non-cognitive traits have also been found to influence performances on cognitive tasks [21,22], we also presented birds with another task to assess their ‘persistence’, in which attempts to acquire clearly visible, but inaccessible, mealworms placed under a Petri dish lid were recorded. As Body Condition has been found to influence performances in PICTs in other birds [23], and differences in growth rates may also influence performances on tasks involving food rewards, we also recorded the Sex and Body Condition of each bird. The repeatability of each individual's performance across the two different PICTs were also determined to assess whether capacities for the same cognitive processes were accurately measured on each task.

## 2. Methods

### (a) Subjects

Two-hundred pheasant chicks were hatched in incubators, randomly assigned to groups of 50 in four replicated enclosures and reared from one day old between 22 May 2017 and 28 July 2017. Eighty-one birds participated in all trials and were included in analyses (see electronic supplementary material, figures S2 and S3).

### (b) Procedures

During testing, birds individually entered an experimental test arena (0.75 × 0.75 m), where they were visually isolated from other birds. For each trial, subjects could acquire a freely available mealworm that was positioned in front of the apparatus (Baseline Worm) and mealworms that were positioned inside the apparatus (Reward Worm). The presentation order of the Barrier and Cylinder tasks was counterbalanced across subjects.

### (c) Detour tasks

#### (i) Opaque training

Each bird initially received four trials on an opaque training apparatus, following MacLean [19]. Subjects were required to learn to detour around a barrier (Barrier task), or reach inside a cylinder (Cylinder task). We recorded latencies (sec) from entering the test chamber to when they acquired the Baseline Worm and Reward Worm, as well as the number of times each bird pecked at the apparatus. A trial ended when the bird acquired the Reward Worm, or after 240 s if they failed to acquire the Reward Worm.

#### (ii) Transparent testing

Birds were presented with three trials on a transparent test apparatus the day after they completed their respective training trials. Details of the Cylinder and Barrier apparatuses are presented in the electronic supplementary material. Test trials were identical to the training trials, except that the apparatus was transparent and the Reward Worm was clearly visible. To access the mealworms, birds had to place their head inside the opening of the pot in the Cylinder task, and detour around the barrier in the Barrier task. Baseline and Reward Worm latencies and Pecks were recorded for each individual.

### (d) Motivation tasks

#### (i) Persistence

Birds were presented with a transparent Petri dish (5 cm diameter), fastened horizontally to a white base (20 × 20 cm), that contained approximately 70 visible, but inaccessible, mealworms. All birds were tested on the same day and experienced one trial in the morning and one trial in the afternoon. We recorded the number of Pecks that each individual directed towards the Petri dish as they attempted to acquire the mealworms for one minute (see electronic supplementary material, figure S1).

#### (ii) Dietary breadth

Before participating in this test, we placed an *ad libitum* supply of commercial parrot food, containing a variety (greater than 10) of different food items (i.e. seeds, dried fruits, chilli peppers, different coloured kibble) in each pen for 7 days. During the testing session, 10 different food items were presented in a fixed array (see electronic supplementary material, figure S1). We recorded how many different food items that each bird sampled within a single 2 min session.

#### (iii) Sex and Body Condition

At 10 weeks old, 2 days after testing had ceased, all subjects were sexed using visual cues and their mass was recorded using a spring balance scale (Slater Super Samsom – precision 5 g). Tarsus length was also measured using a caliper (precision 0.1 mm) to determine Body Condition (mass/tarsus^3^).

### (e) Statistical analysis

We ran separate generalized linear mixed effects models with a poisson error structure for each of our three dependent variables (Baseline Worm latency, Reward Worm latency and Pecks), in R v. 1.1.383 [26] using the lme4 package [27]. For each of our three dependent variables, we also ran three separate models (Models 1–3) based on subsets of our data (i.e. 9 models in total). Using the full dataset, we assessed differences in performance between opaque and transparent apparatuses, as well as performance across trials (Model 1). Using first trial performances on the transparent test apparatuses, we assessed whether performances of experienced birds differed from those of inexperienced birds (Model 2). Experienced birds were those that had previously experienced an inhibitory control task, whereas inexperienced birds had not previously experienced a task. Using first trial performances from the first transparent test apparatus that birds experienced, that is, from inexperienced birds, we assessed whether measures of Persistence, Dietary Breadth, Sex or Body Condition influenced performance (Model 3). All independent variables included in each model are presented in table 1. In each model we included an Observational Level Random Effect (i.e. row number) to control for overdispersion [28]. Repeatability of individual first trial test performances on each task were conducted in the RptR package [29], following [30]. We generated Spearman's correlation coefficients, following previous studies on dogs [8,10], to compare first trial test performances across the two different tasks. These comparisons were restricted to inexperienced birds. For repeatability and Spearman's correlations, we transformed the dependent measures for each task into *Z*-scores to standardize the different scales of performances across tasks. To assess whether individual performances were consistent across tasks, we compared first trial test performances from the first task that birds experienced with those from the second task. We assessed performances on the Persistence task by comparing the number of Pecks each individual made on the first and second trials using a paired *t*-test. Correlations between individual performances on the first and second trials of the Persistence task were also assessed using Spearman's rank correlation coefficients. Spearman's correlation coefficients and *t*-tests were conducted in SPSS [31].

**Table 1.** Generalized Linear Mixed Effects Models (GLMM) of performance measures on the Cylinder and Barrier tasks. Non-significant interactions (NS) were removed from models prior to determining the test statistic.

model	explanatory variables	Baseline Worm latency	Reward Worm latency	Pecks
1	(a) Opaque versus transparent	*X*^2^ = 2.92, *p* = 0.09	*X*^2^ = 72.61, *p* < 0.01	*X*^2^ = 549.07, *p* < 0.01
1	(b) Task (Cylinder)	*X*^2^ = −0.06, *p* = 0.79	*X*^2^ = −372.56, *p* < 0.01	*X*^2^ = −82.91, *p* < 0.01
1	(c) Improvement across trials	*X*^2^ = −10.47, *p* = 0.01	*X*^2^ = −97.10, *p* < 0.01	*X*^2^ = −69.37, *p* < 0.01
2	(a) Experience (yes)	*X*^2^ = −8.26, *p* = 0.004	*X*^2^ = −27.86, *p* < 0.001	*X*^2^ = −14.74, *p* = 0.0001
3	(a) Persistence	*X*^2^ = −1.33, *p* = 0.25	*X*^2^ = 0.65, *p* = 0.42	*X*^2^ = 8.58, *p* = 0.003
3	(b) Dietary breadth	*X*^2^ = −1.50, *p* = 0.22	*X*^2^ = 1.27, *p* = 0.26	*X*^2^ = 5.62, *p* = 0.018
3	(c) Body Condition	*X*^2^ = −0.01, *p* = 0.92	*X*^2^ = 0.91, *p* = 0.34	*X*^2^ = −0.02, *p* = 0.90
3	(d) Sex (male)	*X*^2^ = 1.11, *p* = 0.29	*X*^2^ = −3.08, *p* = 0.08	*X*^2^ = −0.21, *p* = 0.65
3	(e) Sex (male) * Body Condition	n.s.	n.s.	n.s.

## 3. Results

### (a) Do transparent test apparatuses evoke prepotent responses?

**Figure 1.**
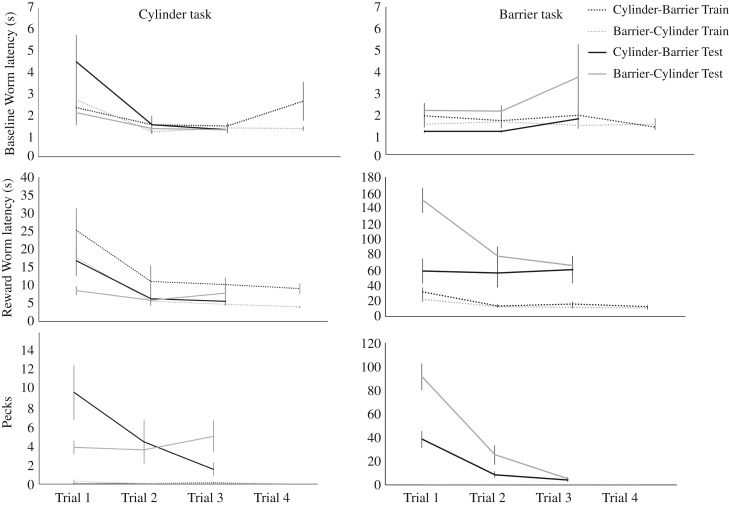
Performances (means ± s.e.) across trials on the Training (opaque) and Test (transparent) Cylinder and Barrier tasks for birds that were Experienced (had previously experienced either the Cylinder or Barrier tasks beforehand) or Inexperienced (had no prior experience on the Cylinder or Barrier tasks).

Baseline Worm acquisition latencies did not differ between opaque and transparent apparatuses. However, birds took longer to acquire the Reward Worm, and made more Pecks, on the transparent test apparatuses compared with the opaque training apparatuses (table 1: Model 1a, figure 1).

### (b) Do performances differ between tasks?

Baseline Worm acquisition latencies did not differ between the Barrier and Cylinder tasks, yet birds took longer to acquire the Reward Worm, and made more Pecks, on the Barrier task, compared with the Cylinder task (table 1: Model 1b, figure 1).

### (c) Do performances improve across trials?

Birds acquired the Baseline Worm and Reward Worm faster, and made fewer Pecks, as trials progressed (table 1: Model 1c, figure 1).

### (d) Does previous experience influence subsequent performance?

Birds that had previously experienced either the Cylinder or Barrier tasks were faster to acquire the Baseline Worm and Reward Worm, and made fewer Pecks on their first test trial of their second task (table 1: Model 2a, figure 1).

### (e) Are individual performances consistent across tasks?

Baseline Worm acquisition latencies were inversely related across tasks (*R*_s_ = −0.335, d.f. = 81, *p* = 0.002), and there was no relationship between Reward Worm latencies (*R*_s_ = 0.181, d.f. = 81, *p* = 0.106) or Pecks (*R*_s_ = 0.122, d.f. = 81, *p* = 0.279) across the two tasks. Individuals showed no repeatability in their Baseline Worm acquisition latencies (*R* = 0.0, s.e. = 0.066, CI = [0, 0.215], *p* = 1), however, this is a likely consequence of low variation in this response measure. Latencies to acquire the Reward Worm were moderately repeatable across tasks (*R* = 0.262, s.e. = 0.108, CI = [0.32, 0.451], *p* = 0.008), but repeatability of Pecks to the transparent apparatuses was low and non-significant (*R* = 0.175, s.e. = 0.102, CI = [0, 0.392], *p* = 0.058).

### (f) Is persistence and dietary breadth related to performance?

Baseline Worm and Reward Worm latencies were unrelated to Persistence or Dietary Breadth (table 1: Model 3a, b). However, birds that made more Pecks on the transparent test apparatuses were also more persistent in pecking at the inaccessible mealworms placed under a clear Petri dish lid, and had a wider Dietary Breadth (table 1: Model 3a, b). An individual's performance in the persistence task was repeatable, suggesting that it reliably captured their persistence. Although birds made fewer Pecks on their second trial of the Persistence task, compared with their first trial (Trial 1: mean = 49.88 ± 2.70 s.e., Range = 0–110; Trial 2: mean = 13.86 ± 1.64 s.e., Range = 0–78): paired *t*-test: *t* = 13.402, d.f. = 80, *p* < 0.001), their number of Pecks correlated positively across trials (Spearman's correlation coefficient): *R*_s_ = 0.33, *N* = 81, *P* = 0.003 (electronic supplementary material, figure S4), and individuals showed high repeatability across trials: *R* = 0.615, s.e. = 0.289, CI = [0, 0.884], *p* < 0.01. Birds sampled an average of 4.10 ± 0.35 s.e. (Range 0–10) different food items on the Dietary Breadth task.

### (g) Does Sex and Body Condition influence performance?

Sex and Body Condition had no influence on Baseline Worm latencies, Reward Worm latencies or number of Pecks (table 1: Model 3c,d).

## 4. Discussion

Pheasants showed inconsistent performances on two different, but functionally identical, tasks that purportedly assess capacities for inhibitory control. Performance on these tasks were instead explained by previous experience with transparent barriers and other non-cognitive behavioural attributes, including greater persistence and a wider dietary breadth. It is unlikely that these findings were due to differences in food motivation, as performance was unrelated to Sex, Body Condition or latencies to acquire a freely available mealworm (Baseline Worm). Our findings therefore raise three fundamental implications that should be considered when using detour tasks to infer capacities for inhibitory control. First, that differences in individual experience must be controlled. Second, that non-cognitive, motivational traits may confound performance on cognitive tasks. Third, that the construct validity of different inhibitory control tasks is unclear and requires further investigation.

Performances differed between transparent and opaque apparatuses for both the Cylinder and Barrier tasks. These findings were not due to novelty, as there were no differences in latencies to acquire a freely available Baseline Worm between transparent or opaque apparatuses. Birds took longer, and made more incorrect attempts, to acquire the food reward on the transparent, rather than opaque, apparatuses. Hence, both the Cylinder and Barrier tasks successfully evoked prepotent motor actions, and required subjects to inhibit these actions to acquire a desired food reward. Like guppies [20], pheasants took longer, and made more errors, to solve the Barrier task compared with the Cylinder task. Again, we consider that their impaired performances on the Barrier task was not due to neophobia, as their latencies to approach and acquire a freely available Baseline Worm did not differ between the two tasks. Consequently, Barrier tasks may be more challenging to solve than Cylinder tasks. Future studies should therefore implement these findings to investigate developmental capacities for inhibitory control in animals, using increasingly difficult tasks as has previously been shown in human infants [32].

As trials progressed on a given task, birds became faster, and made fewer redundant attempts to acquire the reward. Hence, birds learnt to inhibit their prepotent responses as they gained experience of each task. Consequently, an individual's performance on one trial is not independent of their performance on previous trials. Experience should therefore be considered when multiple presentations of such PICTs are made. Moreover, general experience with transparent obstacles, irrespective of the type of apparatus used (barrier or cylinder), also corresponded with improved performance on a subsequent novel task variant. As such, birds that had previously experienced a transparent apparatus made fewer errors on a subsequent PICT compared with inexperienced individuals. However, by counterbalancing the presentation order of tasks, it also remains possible that birds' performances improved on their second task as they were older. Yet as there was only a 3 day interval between their first and second testing sessions we consider this an unlikely explanation. To control for possible age effects, future studies could incorporate a third group that experienced their first trial on a transparent apparatus at the same age as those birds that had previously experienced a transparent apparatus. Our findings therefore raise important implications for using detour tasks to infer capacities for inhibitory control between individuals or species that have received different exposure to transparent barriers. Such findings may be relevant to studies on animals from the wild or in urban environments, as their previous experiences with transparent barriers may be unknown. Moreover, differential experience may be particularly problematic when inferring capacities for inhibitory control in domestic species, such as dogs, that frequently experience glass windows and doors [8]. Indeed, pet dogs have been found to outperform shelter dogs on a similar detour task [11]. We might also expect species that are regularly tested on transparent apparatuses, or behind transparent barriers which separate experimenters from subjects, to show superior capacities for inhibitory control. Such experience may explain why primates, particularly the non-human great apes, initially outperformed corvids on inhibitory control tasks [19], but failed to do so when corvids received modified testing procedures [3,5].

An individual's performance on our PICTs was not only influenced by their prior experience with transparent barriers, but also related to individual differences in other non-cognitive traits. Pheasants showed individual consistency in their attempts to retrieve inaccessible mealworms across trials. Yet, although pheasants generally made fewer Pecks on their second trial on the Persistence task, those birds that were more persistent also showed inferior performances on the PICTs. In contrast to primates [19], however, these birds also appeared to have a wider dietary breadth, as indicated by the greater number of food items sampled in the Dietary Breadth task. It remains unclear why superior capacities for inhibitory control in pheasants should correspond with a narrower dietary breadth, whereas in primates a wide dietary breadth was a strong predictor of species differences in inhibitory control [19]. It is possible that in pheasants, individuals that were highly motivated for food found it more difficult to inhibit prepotent attempts to acquire a visible mealworm. Indeed, North Island robins in poor Body Condition had inferior inhibitory control on a Cylinder task than those birds in good Body Condition [23]. However, we found no evidence that either persistence or dietary breadth influenced latencies to acquire a freely available worm (Baseline Worm), or latencies to solve each task (Reward Worm). Moreover, as males are larger than females [33], we might expect their faster growth rates to result in higher food motivation. Yet performances on PICTs by pheasants were unrelated to Sex or Body Condition. Consequently, the influence of persistence and dietary breadth on an individuals' performance on PICTs suggests that other non-cognitive traits should also be considered when assessing putative cognitive traits.

We found little evidence that an individual's performance was consistent across two similar PICTs suggesting that Barrier tasks have a low construct validity and may not provide a reliable assay of individual capacities for inhibitory control. We found no repeatability of inter-individual variation in the number of incorrect attempts (Pecks) to solve different PICTs. However, inter-individual variation in latencies to solve different PICTs were moderately repeatable. These findings suggest that the number of errors that individuals made on one task were unrelated to their errors made on the other task. However, the speed in which individuals solved each task were consistent, possibly due to similarities in their inherent motivational state. Like studies on dogs that use large sample sizes, we found no evidence that individual performance across multiple inhibitory control tasks was related [8,10,11]. Repeatability of cognitive performance are rarely reported in animals [34]. While such procedures have been considered important in establishing the stability of individual differences in personality traits across time and context [35,36], it remains unclear whether similar processes or mechanisms underlie performance on cognitive tasks. Our findings therefore question whether performance on these two different inhibitory control tasks are mediated by the same processes. Further investigation into the neuromechanisms that influence performance on different inhibitory control tasks is therefore required to validate their use as robust assays of cognition in animals.

In summary, our findings reveal three important implications when inferring capacities for inhibitory control using detour tasks. First, comparisons of inhibitory control performances, between individuals or species, may be confounded by different experiences with transparent barriers. Second, non-cognitive traits, such as persistence or dietary breadth, may contribute to individual differences in inhibitory control performance. Finally, the mechanisms underlying cognitive performance on different inhibitory control tasks remains unclear and requires further investigation. Our findings highlight how non-cognitive traits can influence performance on tasks that are considered to assess particular cognitive capacities.

## Supplementary Material

Methods, pictures of test apparatuses and flow diagram of inhibitory control performance across trials

## Data accessibility

All data are available on Dryad (http://dx.doi.org/10.5061/dryad.qn66gr2) [37].
