# Supplementary material for: Do detour tasks provide accurate assays of inhibitory control?
Source: Proc Biol Sci. 2018 Mar 28;285(1875):20180150. doi: 10.1098/rspb.2018.0150 (PMC5897648; doi:10.1098/rspb.2018.0150)
Supplement: Methods, pictures of test apparatuses and flow diagram of inhibitory control performance across trials [file rspb20180150supp1.docx]

**Electronic Supplementary Material:** *Do detour tasks provide accurate assays of inhibitory control?*

*Proceedings of the Royal Society: B*

Jayden O. van Horik*, Ellis J.G. Langley, Mark A. Whiteside, Philippa R. Laker, Christine E. Beardsworth and Joah R. Madden

Centre for Research in Animal Behaviour, Psychology, University of Exeter, UK

*Correspondence: Jayden O. van Horik

E-mail address: jayden.van.horik@gmail.com

**Supplementary Methods**

**Subjects**

Birds were individually marked using numbered wing tags, fed on commercial pheasant feed (Keepers’ Choice) supplied with water *ad libitum*. Birds were housed in 2m x 2m heated pens for the first 2 weeks of life. They had access to unheated, but covered outdoor runs of 1m x 4m for the next week. For the final seven weeks of rearing each group had additional access to separate 4m x 12m outdoor enclosures. Aside from the tasks reported in the current study, birds were also tested on a battery of spatial cognition and discrimination learning tasks from 17 days old; with equal exposure in a fixed order to all tasks.

**Procedures**

Birds were habituated to human observation from one day old. Shaping procedures, using meal-worm rewards, were adopted to habituate subjects to the testing arena. Each test arena was artificially illuminated by a single 20w domestic lightbulb between 0800-18:00. On any given task, subjects were first presented with an opaque training apparatus and then proceeded to a transparent test apparatus. Birds participated on their first training session on 13 July 2017 and their first test session on 14 July 2017. Birds then were trained on their second apparatus on 17 July 2017, and tested on the second apparatus on 18 July 2017. All subjects received their four training trials, for a given task, on the same day. The purpose of the training trials was to ensure that subjects possessed the correct motor-skills to successfully navigate the task and acquire the reward. Baseline Worm acquisition latencies were used to determine any effects of neophobia and to ensure that subjects were food-motivated. Birds from two enclosures began with the Barrier task and proceeded with the Cylinder task, while the remaining birds from the other two pens began with the Cylinder task and proceeded with the Barrier task. After completing both tasks, on 19 July 2017, subjects participated in a Persistence task (between approximately 08:00-12:00) and then later on the same day a Dietary Breadth task (between approximately 14:40-16:15).

**Inclusion/Exclusion of Subjects for Analyses**

Birds that failed to acquire both the Baseline Worm and Reward Worm within 240 sec on any given trial were excluded from the current study. Birds that acquired the Baseline Worm and made at least one peck to the apparatus, but failed to acquire the Reward Worm within 240 sec, were given a ceiling value of 300 sec.

Eighty-one subjects participated in all trials of all tasks (a total of 17 trials per bird). Of these birds, 51 (32 Male, 19 Female) fist experienced the Barrier task and the proceeded to the Cylinder task, while 30 birds (20 Male, 10 Female) first experienced the Cylinder task and then the Barrier task. All birds retrieved the Baseline Worm and Reward Worm within 240 sec on the Cylinder task training and test trials, and on the Barrier task training trials. However, while the following birds acquired the Baseline Worm and pecked at least once at the Barrier task test apparatus, they failed to retrieve the Reward Worm within 240 sec. These birds were included in the study but given Reward Worm latencies of 300 sec. The number of trials that birds received Reward Worm latencies of 300 sec in the Cylinder-Barrier condition were as follows: one bird failed on all three trials; three birds failed on two trials; two birds failed on one trial. In the Barrier-Cylinder condition: one bird failed all three trials; 6 birds failed on two trials; 11 birds failed on one trial.

**Detour Tasks**

*Cylinder*

The Cylinder task was a modified plastic pot with an opening at one end (5cm diameter). A stand (9 cm high) was fixed to the closed end of the pot and fastened to a base (20c x 20cm). Approximately 10 mealworms were placed inside the pot, but positioned behind a lip so that they could not escape. In all cases the mealworms were clearly visible from the opening of the pot. Birds had to place their head inside the opening of the pot to access the mealworms.

*Barrier*

The Barrier task was comprised of two plastic Perspex sheets (25cm wide x 100cm high) that were fixed together at right angles so that they formed an “L” shape. The apparatus was positioned against the wall of the test arena, prohibiting access on a third side. Approximately 10 mealworms were placed inside a white lid (5cm diameter), which was positioned behind the barrier. To access the mealworms the subject had to detour around the barriers.

**Supplementary Ethics**

These procedures were considered to mitigate stress and encouraged subjects’ voluntarily participation during testing. Birds could therefore choose whether to participate in tasks. There were no enforced aversive stimuli. Birds that failed to engage with the task in 240 secs were permitted to pass into the recovery area and their lack of participation recorded. Birds were reared at a lower density than that recommended by DEFRA’s code of practice (DEFRA, 2009), thus reducing likely stress and competition between chicks.

**Supplementary Figures**


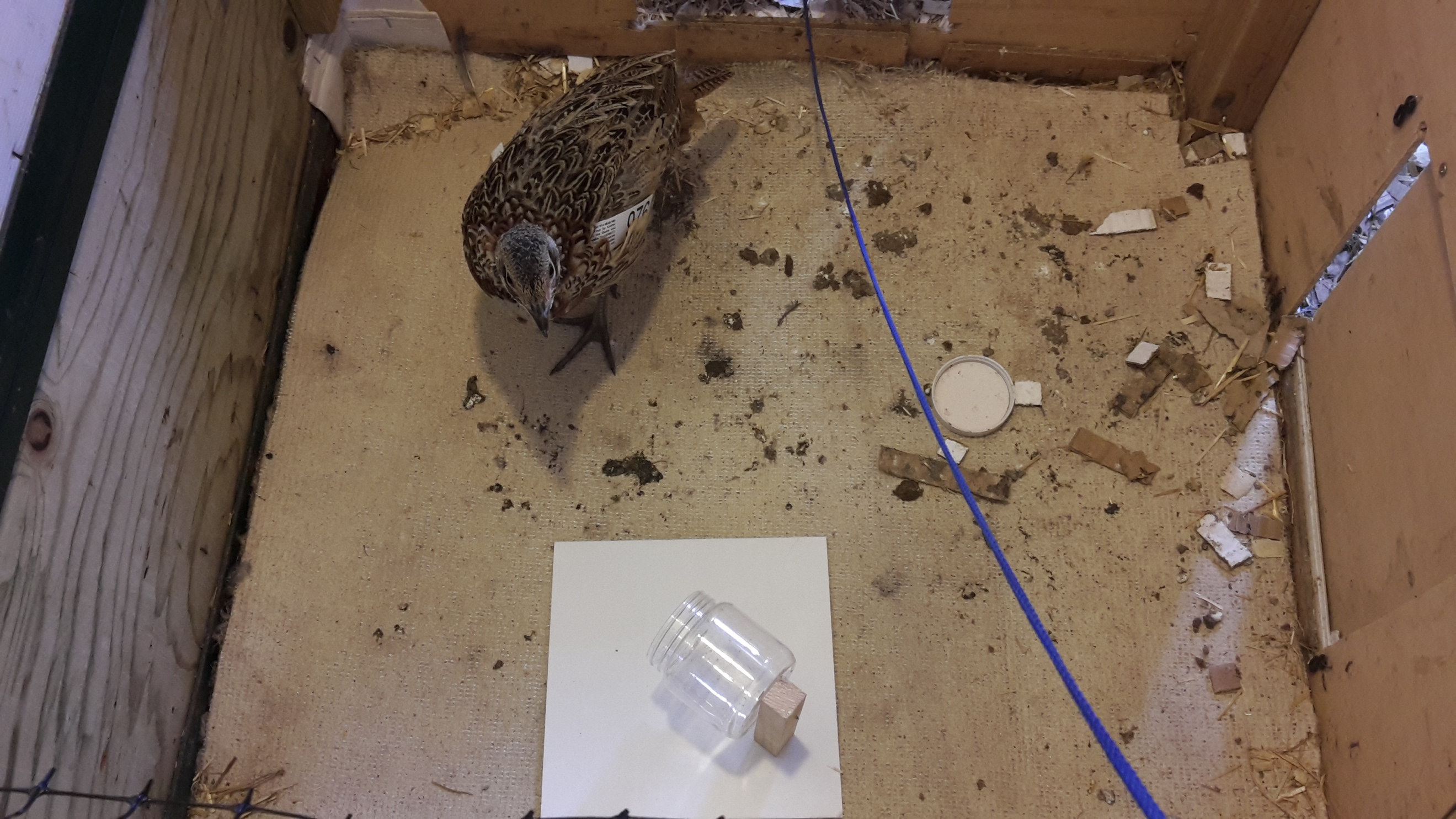

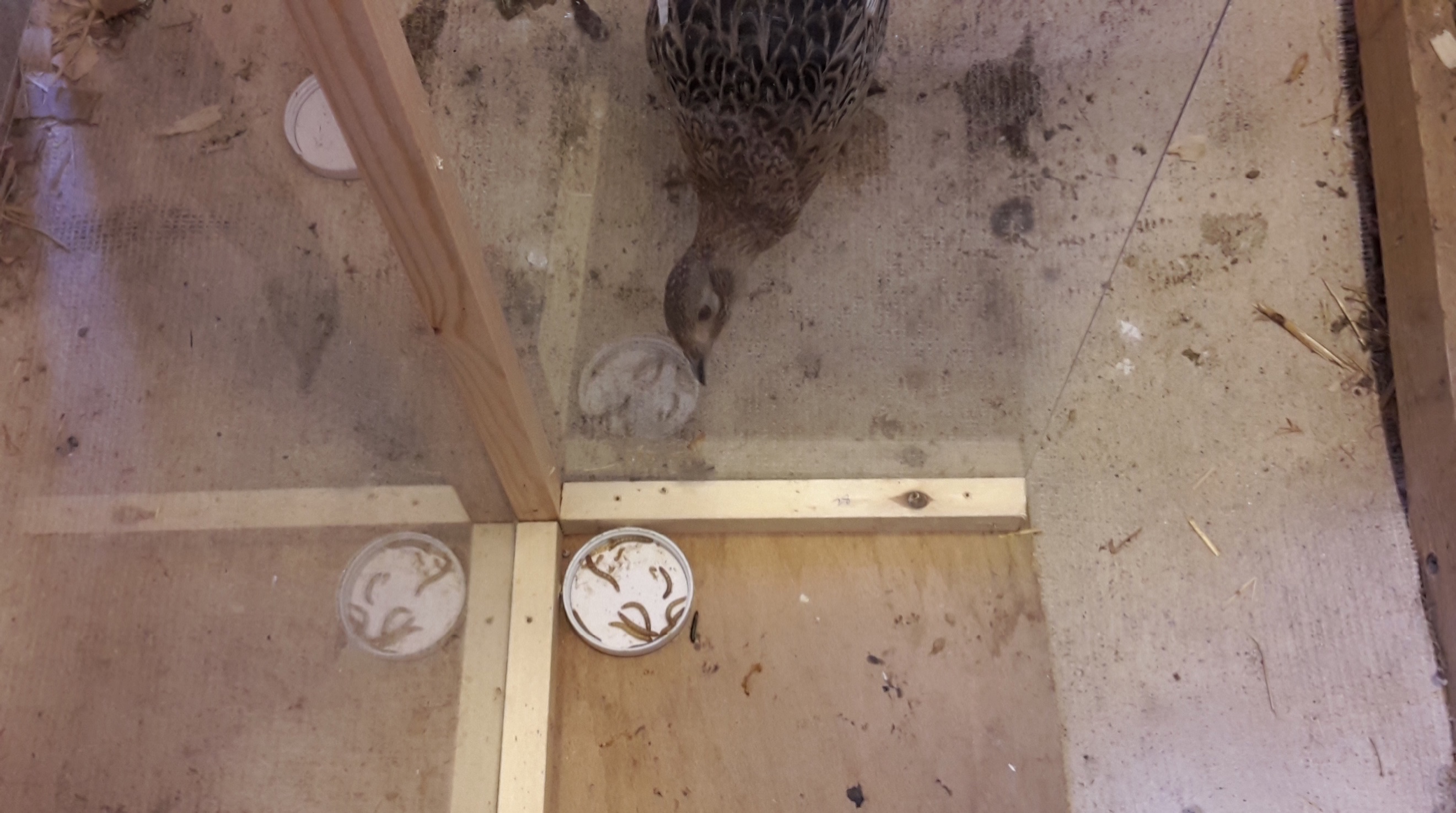

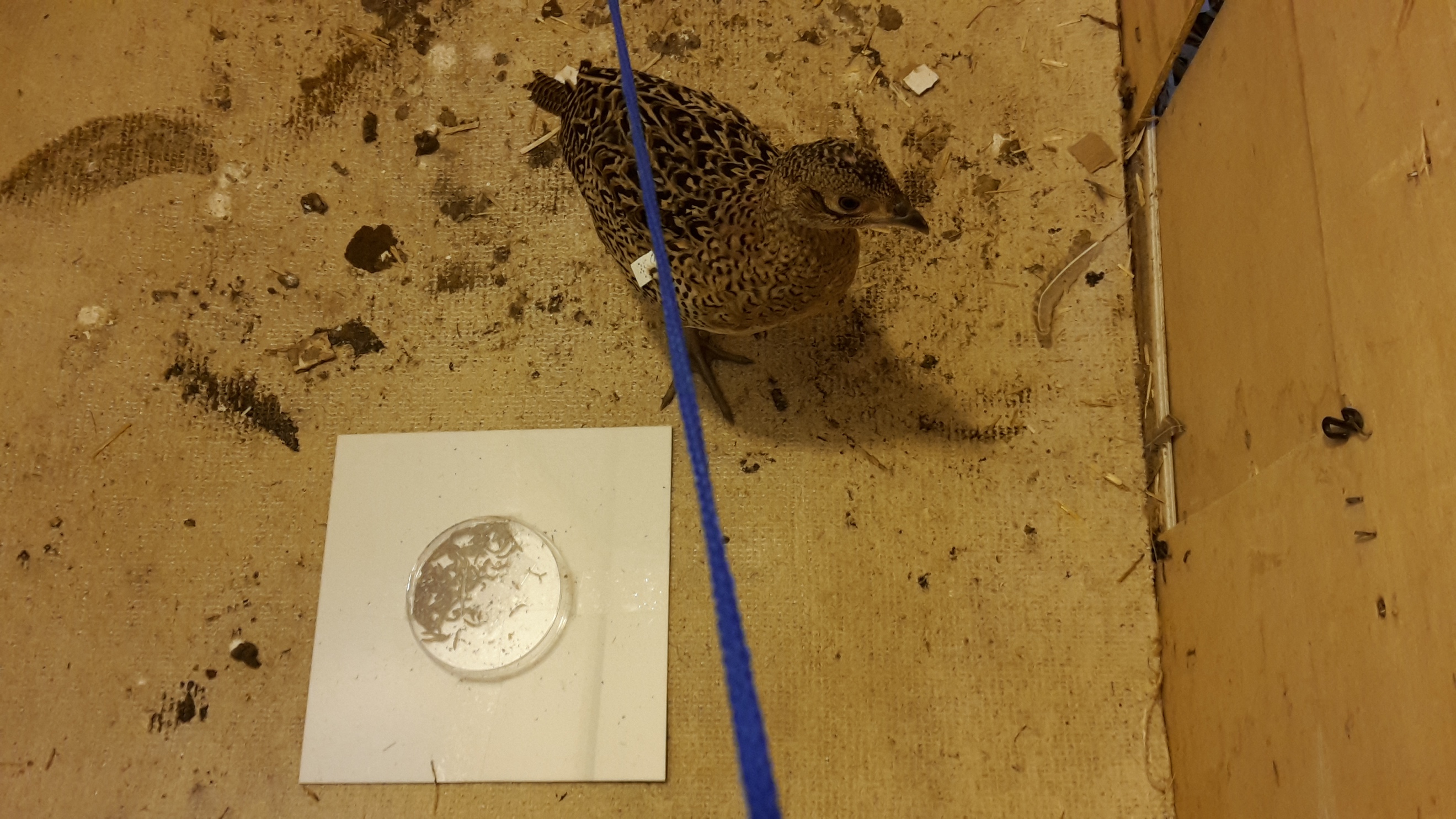

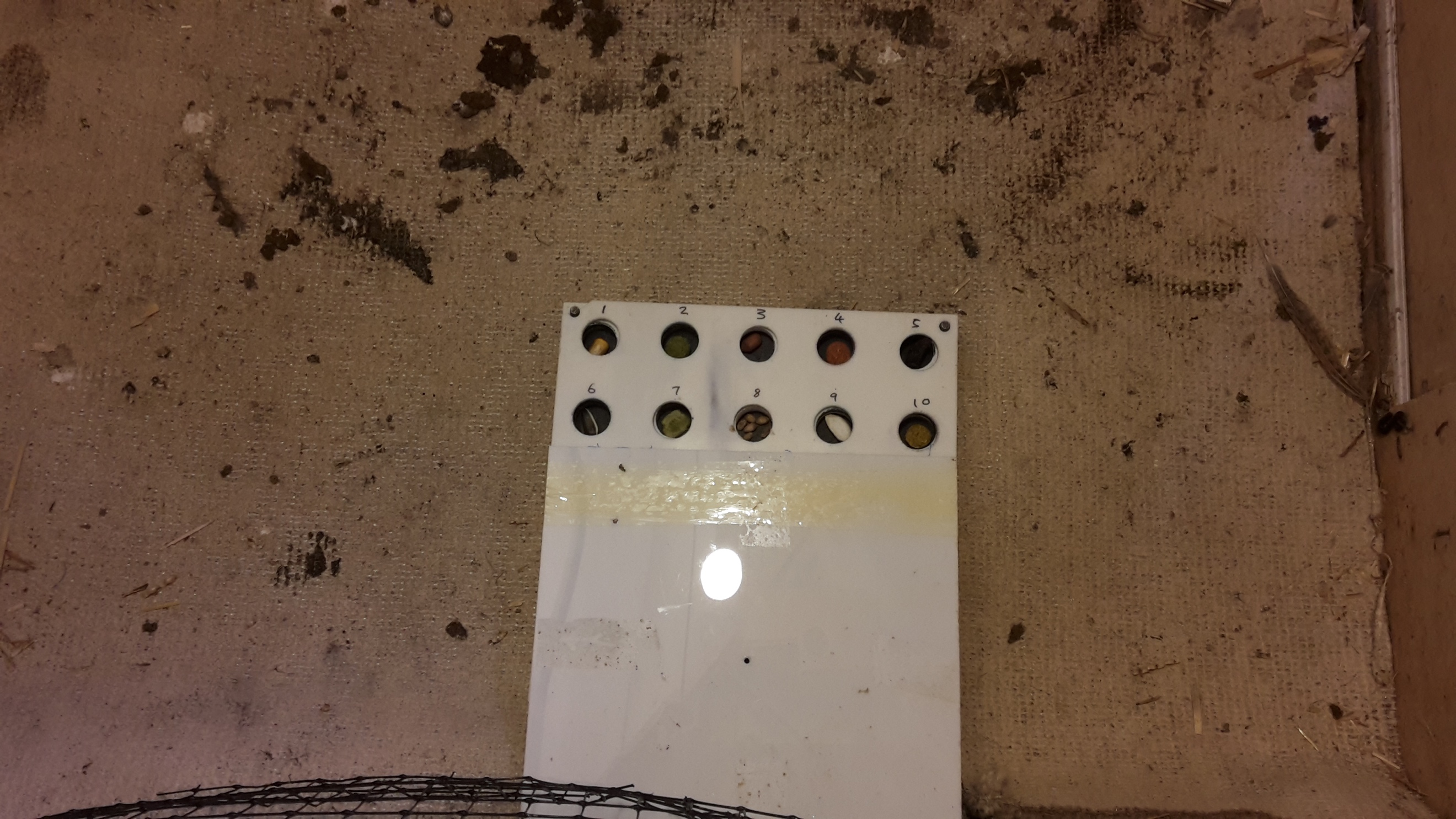


**Figure 1**. Left: Transparent Barrier (top) and Cylinder (bottom) test apparatuses used to assess inhibitory control performance. Right: Food choice (top) and Persistence (bottom) tasks used to assess food motivation.

**Figure 2a.**

**Figure 2b.**

**Figure 3a.**

**Figure 3b.**

**Figures 2a, 2b, 3a, 3b.** Performance of pheasants on the Cylinder and Barrier tasks across trials. Successful birds made no errors (pecks) and hence directly acquired the mealworm reward. Unsuccessful birds made at least one error prior to acquiring the reward. CB group first participated in the Cylinder task and then the Barrier task. BC group first participated in the Barrier task and then the Cylinder task.


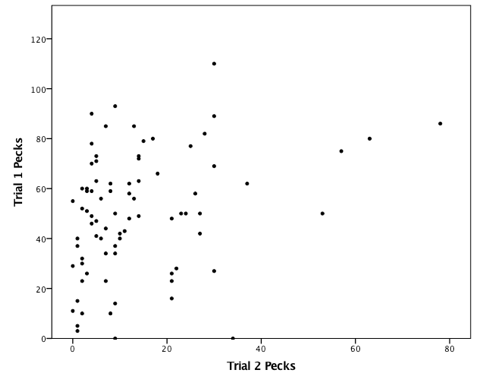


**Figure 4.** Performances on the Persistence task. Number of pecks each individual made while attempting to acquire inaccessible mealworms placed under a Petridish in one minute.

**References**

DEFRA. (2009). Code of practice for welfare of game birds reared for sporting purposes.
